# Supplementary material for: Patterns of Intron Gain and Loss in Fungi
Source: PLoS Biol. 2004 Nov 30;2(12):e422. doi: 10.1371/journal.pbio.0020422 (PMC532390; doi:10.1371/journal.pbio.0020422)
Supplement: Table S1 — Also available at http://genes.mit.edu/NielsenEtAl/. (4.3 MB ZIP). [file pbio.0020422.st001.zip › NielsenEtAl/html/350.html]

AN6492.1.NCU00116.1.MG00472.1.FG02608.1


```
 CLUSTAL W (1.82) Multiple Sequence Alignments - Introns Inserted


Sequence 1: NCU00116.1	260 aa
Sequence 2: MG00472.1	270 aa
Sequence 3: FG02608.1	277 aa
Sequence 4: AN6492.1	269 aa
Alignment Length: 293 aa
Number Identitical Residues: 136 aa
Alignment Score (without introns) 6123


MG00472.1 	MAMNPHDATQAQAQGQAQDGRPQANYDLSKGGHYG1ASSQ0LAQQG~YAPS-EHYTGPWA
NCU00116.1	--MDPQNPSSA---GHNQQQRQQPVYDTSQGGHY-~----~----G1FAPS-DLYTGTWA
FG02608.1 	--MDPQNSQAA------QQSRQRPVYDPSQGGHYG1ASAQ0LASQG~FAPS-ELYTGPWA
AN6492.1  	--MEQAQQTSG-----QQQGRQQPVYDTRQGGHYG1ASAA0LAAQG~FAPVAELYTGTWA
          	  *:  :   .      *: * :. **  :****. :::   : .* :** :: ***.**

MG00472.1 	N~VHQGLKGPYKNILTTYWQHTIDHLENDTHDYKLHQLPLARIKKVMKADPEVKMISAEA
NCU00116.1	N~VHQGLQGNYKEILTAYWQQTINHLENDTHDYKLHQLPLARIKKVMKADPEVKMISAEA
FG02608.1 	N0AHQGLTGQYKDILTTYWQHTISHLENDTHDYKIHQLPLARIKKVMKADPEVKMISAEA
AN6492.1  	N0VNQGLQGTARDILTTYWQHVINHLESDNHDYKIHQLPLARIKKVMKADPEVKMISAEA
          	* .:*** *  ::***:***:.*.***.*.****:*************************

MG00472.1 	PILFAKGCDIFITELTMRAWIHAEENKRRTLQRSDIASALSMSDMFDFLIDIVPREEAAG
NCU00116.1	PILFAKGCDIFITELTMRAWIHAEENKRRTLQRSDIASALSKSDMFDFLIDIVPREEAST
FG02608.1 	PILFAKGCDIFITELTMRAWIHAEENKRRTLQRSDIASALAKSDMFDFLIDIVPREEASS
AN6492.1  	PILFAKGCDVFITELTMRAWIHAEDNKRRTLQRSDIAAALSKSDMFDFLIDIVPREEATS
          	*********:**************:************:**: ****************: 

MG00472.1 	HAKRAAGQAGAANAAAGQ---PQMGGMP---AQNHT-QH-MGA-EYGLPGHPMGGEADYR
NCU00116.1	HAKRATGQSAAAAAQAAQNAAQQAQGMPGVAAPNHGGQHPMAAPDYTLGGHAIAPEADYR
FG02608.1 	HAKRATQTAAAQPLPAGQ--AQMPGQHPMAQAPNPG--HPMGA-EYMPS-HGIAPEQDYR
AN6492.1  	HAKRSSQSAGAPAGPGGP---TAAGQLPQTQHGVQHHPHHMAPPDYGALGQHPLQDQEYR
          	****::  :.*    ..          *          * *...:*   .:    : :**

MG00472.1 	QAQNMYPGQVPTGAPNAYGQPQQP---MYEVDPNMYSSYTMQPPQ0S~A----------
NCU00116.1	Q-QNMYSGQVQPGAPAPYGQPQPP---MYDVDG-MY--YSTMPPQ~Q0-----------
FG02608.1 	NPANMYPDQSVPNPQAAYGQAQQPNMNPYGAMGDMYP-YPGMAPQ~Q0TTMSNEEFGEQ
AN6492.1  	Q-QTMYGGAVQSDPTAAYAQPQTQ------MFEGMYTAYPHLPPQ~Q~VRIG-------
          	:  .** .   ...  .*.*.*           .**.:*.  .** .    .
```
